# Body surface infrared thermometry in patients with central venous cateter-related infections

**DOI:** 10.1590/S1679-45082015AO3397

**Published:** 2015

**Authors:** José Henrique Silvah, Cristiane Maria Mártires de Lima, Maria do Rosário Del Lama de Unamuno, Marco Antônio Alves Schetino, Luana Pereira Leite Schetino, Priscila Giácomo Fassini, Camila Fernanda Costa e Cunha Moraes Brandão, Anibal Basile, Selma Freire Carvalho da Cunha, Julio Sergio Marchini

**Affiliations:** 1 Hospital das Clínicas, Faculdade de Medicina de Ribeirão Preto, Universidade de São Paulo, Ribeirão Preto, SP, Brazil.; 2 Universidade Federal de Minas Gerais, Belo Horizonte, MG, Brazil.

**Keywords:** Central venous catheters, Catheter-related infections, Thermometry, Infrared rays, Early diagnosis, Intensive care

## Abstract

**Objective:**

To evaluate if body surface temperature close to the central venous catheter insertion area is different when patients develop catheter-related bloodstream infections.

**Methods:**

Observational cross-sectional study. Using a non-contact infrared thermometer, 3 consecutive measurements of body surface temperature were collected from 39 patients with central venous catheter on the following sites: nearby the catheter insertion area or totally implantable catheter reservoir, the equivalent contralateral region (without catheter), and forehead of the same subject.

**Results:**

A total of 323 observations were collected. Respectively, both in male and female patients, disregarding the occurrence of infection, the mean temperature on the catheter area minus that on the contralateral region (mean ± standard deviation: -0.3±0.6°C* versus *-0.2±0.5ºC; p=0.36), and the mean temperature on the catheter area minus that on the forehead (mean ± standard deviation: -0.2±0.5°C* versus *-0.1±0.5ºC; p=0.3) resulted in negative values. Moreover, in infected patients, higher values were obtained on the catheter area (95%CI: 36.6-37.5ºC *versus* 36.3-36.5ºC; p<0.01) and by temperature subtractions: catheter area minus contralateral region (95%CI: -0.17 - +0.33ºC *versus* -0.33 - -0.20ºC; p=0.02) and catheter area minus forehead (95%CI: -0.02 - +0.55ºC *versus* -0.22 - -0.10ºC; p<0.01).

**Conclusion:**

Using a non-contact infrared thermometer, patients with catheter-related bloodstream infections had higher temperature values both around catheter insertion area and in the subtraction of the temperatures on the contralateral and forehead regions from those on the catheter area.

## INTRODUCTION

Central venous catheters (CVC) are devices that allow delivery of medication and nutritional therapies as well provide hemodynamic measures that cannot be safely accomplished otherwise.^1^These vascular accesses can be classified as short and long-term use. Short-term CVC are usually non-tunneled polyurethane catheters with single or multiple lumens and should be inserted only in hospitalized patients to use for days or weeks.^2^ Examples of long-term CVC (>3 months) are the cuffed tunneled catheters (*i.e.,* HICKMAN^®^ or BROVIAC^®^) and the totally implantable Port. The last one is preferred for patients who require intermittent vascular access.^3^

Near 5 million CVC are inserted per year, in the United States, with a documented rate of related infections ranging between 3 to 26% per year.^4-7^ Considering only intensive care units, 80 thousand of catheter-related bloodstream infections (CRBSI) occurred in a year.^8^ The costs related to each case of CRBSI could reach US$56,000,^9^ with an average increase in hospital stay of 6.5 days for critically ill patients.^10^ In addition, CRBSI have been associated with up to 25% mortality,^8,10^ catheter replacement and interruption of intravenous therapy.^11,12^

Researchers had described a low sensibility to detection of local inflammation around CVC insertion area based on clinical findings (pain, erythema, swelling and purulence).^13^ They also reported that vascular device colonization could happen even in the absence of infectious symptoms or signs.^14,15^ On the other hand, the body surface temperature, evaluated with a non-contact infrared thermometer (NCIT), could be altered nearby infectious processes.^16^

Because of that, seems to be reasonable to hypothesize that the NCIT could be helpful to the earlier diagnosis of CRBSI.

## OBJECTIVE

To evaluate if body surface temperature around the central venous catheter insertion area is different when patients develop catheter related infections, when evaluated by a non-contact infrared thermometer.

## METHODS

### Design and patients

This was an observational cross-sectional study aimed to describe the body surface temperature measured with a NCIT, near to where non-tunneled CVC were inserted or the totally implantable CVC reservoir was located. The inclusion criteria were need of a CVC for medical care and be an inpatient of clinical ward or intensive care unit of a public university hospital. The exclusion criteria comprised current treatment of a CRBSI in the beginning of data collection. The study was approved by the Ethical Review Board of this institution and all subjects or their relatives signed an Informed Consent Form.

### Outcome measures

A NCIT was used (Extech, model IR200^®^, Waltham, Massachusetts, United States), with emissivity fixed in 0.95, accuracy of ±0.3°C (±0.5°F) and 0.1°F/°C resolution, capable to measure body temperature from 32 up to 42.5°C (89.6 to 108.5°F). At least 15 minutes after bath, mobilization, warm or cold compresses, measurements were obtained on body surface of the patients, on the following areas: (1) close to non-tunneled CVC insertion or totally implantable CVC reservoir; (2) at the equivalent contralateral region; and (3) at the forehead. To avoid one single measurement resulting in outliers, three consecutive measurements were taken from each aforementioned area and the mean value was recorded. The same researcher performed all measurements. During measurements, the distance between the NCIT and the patient skin was of 10cm. The area aimed was within a ray of 3cm, medially to the catheter insertion or totally implanted catheter reservoir. All measurements were collected until removing of the catheter without knowledge about infection status of patients. Moreover, diagnosis of CRBSI was confirmed through review of medical records after patient discharge or death.

CRBSI was suspected in case of fever, chills and hypotension and absence of hypovolemia or heart disease.^17^ Thus, in all suspected patients, diagnosis was established according to laboratorial criteria,^18^ confirmed by positive quantitative culture of the catheter’s tip or simultaneous quantitative blood cultures drawn through the CVC and peripheral vein.^17^

### Statistical analyses

Initially, comparisons among observations from patients with and without CRBSI were performed.

Later, for patients with suspected CRBSI, the data that were collected within a range of 3 days before and 1 day after the positive blood cultures were associated with CRBSI. Then, comparisons between those with confirmed CRBSI *versus* those in which CRBSI was ruled out were performed.

Descriptive statistics as mean, standard deviation (SD), range (minimum - maximun), and percentage (%) were used to assess the characteristics of the patients or of the observations. Since this was the first study to explore the use of a NCIT in CRBSI, we choose to describe the findings according to sex. Normal distribution of data was verified by normal probability plots, and outliers were detected by boxplots. Data with normal distribution had 95% confidence intervals (95%CI) disclosed. The Wilcoxon rank-sum test and Student’s t-test were used, as appropriate. All reported p values are two-sided. The analyses were conducted with STATA/IC, version 11.2 (StataCorp LP, United States). Statistical power (1-β) was determined with G*POWER^®^, version 3.1.9.2 (Universität Kiel, Germany).^19^ For all analyses the level of significance was fixed at 5%.

## RESULTS

### Descriptive data

Between August and November 2011, data of 39 patients were collected. The mean age was 57 years [SD: ±17 (16-87); 95%CI: 51-63] for both sexes. As to males, it was 58 years old [SD: ±17 (16-78); 95%CI: 50-65] and 57 years old for females [SD: ±19 (19-87); 95%CI:47-67]. Other characteristics of the subjects are displayed on table 1.

**Table 1:** Characteristics of the 39 volunteers

Variable	n (%)
Age, years	
≤65	22 (56)
>65	17 (44)
Sex	
Male	23 (59)
Female	16 (41)
Hospital unit	
Clinical	21 (54)
ICU	18 (46)
CVC type	
Totally implantable CVC	9 (23)
Non-tunneled CVC	30 (77)
Number of lumens	
One	10 (26)
Two	27 (69)
Three	2 (5)
Preferred veins for CVC insertion	
Right subclavian	15 (38)
Right internal jugular	15 (38)
Others	9 (24)
Most common use for CVC	
Only TPN	10 (26)
TPN/fluids/medications	10 (26)
Others	19 (48)

ICU: intensive care unit; CVC: central venous catheter; TPN: total parenteral nutrition.

Considering the mean values of the three measurements performed on each area, a total of 323 observations were collected from the patients: 192 measurements (59%) from male and 131 (41%) from female subjects. Although patients’ distribution was similar in clinical ward and intensive care unit, approximately 75% of all measures were obtained from clinical patients and 25% from those in the intensive care unit (Table 1). Three quarters of all data came from subjects younger than 65 years and one quarter from patients older than that. In 55% of observations, a single lumen CVC was present, and in 43%, a double-lumen CVC. Five measurements (close to 2%) were performed in two patients with triple-lumen catheters.

This distribution was not similar in both sexes, and double-lumen catheters were placed mostly in females (60%). Similarly, in general, the number of measurements related to patients with non-tunneled CVC and totally implantable CVC, respectively, was distributed into almost equal proportions (49% and 51%), but differently when the analysis was conducted by sex (42% and 58% in males and 59% and 41% in females). Since our hospital is a reference unit to treat patients with intestinal failure, up to 65% of all data was collected in patients with short bowel syndrome. Those patients are admitted with higher frequency to receive total parenteral nutrition (TPN) because home parenteral nutrition is not available in the Brazilian public health system.

The temperatures at catheter area (totally implantable reservoir as well), at the contralateral region and at the forehead were different between sexes (Table 2). Furthermore, the subtraction of temperatures of catheter area minus those of the contralateral side resulted in mean negative values in males and females, respectively (mean±SD: -0.3±0.6 *versus* -0.2±0.5°C; p=0.36). Similar finding was observed by subtracting the temperature of forehead from those of catheter area (mean±SD: -0.2±0.5 *versus* -0.1±0.5°C; p=0.3).

**Table 2:** Temperature measurements according to sex

	Male	Female	p value^*^
	(n=192)	(n=131)	
Temperature area (ºC)			
CVC area	36.4±0.7 (34.9 - 38.2)	36.5±0.8 (34.5 - 39]	0.01
Contralateral region	36.6±0.6 (32.2 - 38.3)	36.8±0.7 (34.5 - 38.2)	0.02
Forehead	36.5±0.5 (35.2 - 38.1)	36.7±0.6 (34.3 - 37.9)	<0.01

Subtractions (ºC)			
CVC – contralateral	-0.3±0.6 (-1.9 - +3.1)	-0.2±0.5 (-1.5 - +1.3)	0.36
CVC – forehead	-0.2±0.5 (-1.7 - +1.3)	-0.1±0.5 (-1.5 - +1.4)	0.3

Data expressed in mean ± standard deviation; range (minimun - maximun). ^*^p values were calculated with Wilcoxon rank-sum test and Student’s* t*-test, as appropriate (α=5%). CVC: central venous catheter.

### Main study results

Four patients, two from each sex, had six cases of infection related to CVC during the study (two patients had two cases). All were younger than 65 years old, none was at the ICU, and three had a totally implantable CVC. The pathogens identified were *Acinetobacter junii*, methicillin-resistant *Staphylococcus aureus* and *Staphylococcus warneri*. Approximately 5% (n=15) of the observations were collected within a range of 3 days before and one day after that positive blood cultures were drawn. Discarding two observations (outliers) and comparing the remaining measurements with the observations not related to CRBSI, higher values were encountered in patients with infection in both subtractions of temperature: catheter area minus contralateral region (95%CI: -0.17 - +0.33 *versus* -0.33 - -0.20°C; p=0.02; statistical power of 0.82), and catheter area minus forehead (95%CI: -0.02 - +0.55 *versus* -0.22 - -0.10°C; p<0.01; statistical power of 0.88), respectively. Moreover, the temperature from catheter area was higher in patients with CRBSI (95%CI: 36.6-37.5 *versus* 36.3-36.5°C; p<0.01; statistical power of 0.94) (Table 3).

**Table 3:** Temperature (ºC) in the observations related and unrelated to infection

	Related to infection		Unrelated to infection		p value^*^
	(n=13)		(n=308)		
	Means±SD	95%CI	Means±SD	95%CI	
CVC area	37.1±0.7	36.6 - 37.5	36.4±0.7	36.3 - 36.5	<0.01
Contralateral region	37.0±0.6	36.6 - 37.3	36.7±0.7	36.6 - 36.8	0.12
Forehead	36.8±0.6	36.4 - 37.1	36.6±0.6	36.5 - 36.6	0.20
CVC – contralateral	+0.08±0.42	-0.17 - +0.33	-0.27±0.54	-0.33 - -0.2	0.02
CVC – forehead	+0.27±0.48	-0.02 - +0.55	-0.16±0.52	-0.22 - -0.1	<0.01

^*^p values were calculated with Student’s *t*-test (α=5%). SD: standard deviation; 95%CI: 95% confidence interval; CVC: central venous catheter.

Among the 308 observations not associated with positive blood cultures, 16 were collected in presence of clinical signs suggestive of infection, between 3 days before and 1 day after withdrawal of negative blood cultures. Therefore, when the measurements related to negative and positive blood cultures were compared, the temperature around the CVC insertion area was higher in those with CRBSI (p=0.03; statistical power of 0.58). Also, higher mean values were encountered in patients with infection in both subtractions of temperatures: catheter area minus forehead (p=0.03; statistical power of 0.85) and catheter area minus contralateral region (p=0.02; statistical power of 0.45), respectively (Table 4).

**Table 4:** Temperature (ºC) in the observations related to blood cultures

	Positive blood cultures	Negative blood cultures	p value^*^
	(n=13)	(n=16)	
	Means±SD	Means±SD	
CVC area	37.1±0.7	36.5±0.5	0.03
Contralateral region	37.0±0.6	36.7±0.2	0.21
Forehead	36.8±0.6	36.7±0.2	0.16
CVC – contralateral	+0.08±0.42	-0.27±0.48	0.02
CVC – forehead	+0.27±0.48	-0.22±0.46	0.03

*p values were calculated with Wilcoxon rank-sum test (α=5%). SD: standard deviation; CVC: central venous catheter.

## DISCUSSION

The NCITs were introduced in some airports and gathering areas during the emergence of the severe acute respiratory syndrome (SARS), in 2003, with reports of a low efficacy.^20^ At the same time, some studies suggested that the NCIT could be a reliable alternative for body temperature measurement, mainly in pediatric patients.^21-23^ Its principle is that heat emitted by the body can be detected in the infrared radiation spectrum through a remote sensor.^24^

As far as it is known, this is the first study about infrared thermometry and catheter related infections. During data collection, six cases of CRBSI were confirmed in four patients, mainly in those with short bowel syndrome who received TPN and other medications or fluids through a totally implantable CVC. Two of the 15 observations related to positive blood cultures generated pronounced negative results (outliers) from the subtraction of temperatures of contralateral region from those of CVC area (-1.3°C and -1.4°C). When the two measurements were taken, nothing was being infused through the catheter, and the patient was receiving a lock therapy with 70% ethanol through it.^25^ However, there is no guarantee of its relation with the results obtained. After exclusion of these two measurements, differences were observed between the subtractions of temperatures from CVC area minus those of contralateral region and forehead, comparing the data related and not with CRBS. The difference observed only around the CVC insertion area of infected patients indicates that, when CRBSI occurs, the body surface temperature, measured with a NCIT, around the CVC insertion area or its reservoir, becomes higher.

When the 13 observations from 3 days before and 1 day after positive blood cultures were compared with the 16 measurements of the same range but after negative blood cultures, similar results were observed.

Among patients with suspected infection, a low statistical power was encountered comparing the values from patients with positive blood cultures to those with negative cultures, regarding to CVC area and the subtraction CVC area minus contralateral region. The small number of observations related to blood cultures could be the explanation.^26^

Recently, studies have highlighted diagnostic and prognostic uses of temperature measurements for infectious diseases. Abnormal patterns of body temperature, evaluated by common thermometers, were disclosed as predictive of sepsis.^27^ Also, skin surface temperature measurements by a NCIT seem to be a promising indicator of severity or improvement of skin and soft tissue infection.^16^

In general, even not considering the occurrence of infection, negative results were observed when the temperatures of contralateral and forehead regions were subtracted from those obtained on the catheter/reservoir area. A lower temperature at the catheter area could be explained by the almost continuous infusion of fluids (*i.e.,* parenteral nutrition and saline solution), which usually are kept bellow body temperature. An infusion of 2L of crystalloid in room temperature decreases body heat by one-third, in Celsius degrees.^28^ However, this assumption should be tested in future studies.

Regarding the study limitations, the number of observations was not equally obtained among the patients, due to three reasons: (1) the hospitalization time varied between them due to death, improvement or worsening of the condition; (2) the data collection was unavailable for some patients in a certain day due to the performance of procedures; (3) the temperature measurements were done only in week days. Thus, close to 41% of the observations were related to only 3 patients, while another 18 subjects contributed with less than 1% of all. In addition, changes in body heat caused by the temperature of the fluid infused at the moment of data collection were not investigated. Finally, this study did not evaluate severity of disease, environment temperature and hemodynamic stability influences on the results, as well if body surface temperature around specific areas is different for each type of vascular device.

Further studies must confirm these findings. Moreover, larger samples seem to be required to detect differences among patients suspected of having infection, with a higher statistical power.^29^ Finally, diagnostic accuracy^30^ should be addressed.

## CONCLUSION

In conclusion, using a non-contact infrared thermometer, patients with catheter-related bloodstream infections had higher temperature values at central venous catheter insertion area and through subtractions between catheter area, contralateral and forehead regions temperatures. Further studies must confirm these findings.
