# Supplementary material for: Body surface infrared thermometry in patients with central venous cateter-related infections
Source: Einstein (Sao Paulo). 2015 Jul-Sep;13(3):364–9. doi: 10.1590/S1679-45082015AO3397 (PMC4943780; doi:10.1590/S1679-45082015AO3397)
Supplement: Translation

# Translation: Termometria infravermelha da superfície corporal em pacientes com infecção relacionada a cateter venoso central

**Authors:** José Henrique Silvah, Cristiane Maria Mártires de Lima, Maria do Rosário Del Lama de Unamuno, Marco Antônio Alves Schetino, Luana Pereira Leite Schetino, Priscila Giácomo Fassini, Camila Fernanda Costa e Cunha Moraes Brandão, Anibal Basile, Selma Freire Carvalho da Cunha, Julio Sergio Marchini

**Affiliations:** 1 Hospital das Clínicas, Faculdade de Medicina de Ribeirão Preto, Universidade de São Paulo, Ribeirão Preto, SP, Brasil.; 2 Universidade Federal de Minas Gerais, Belo Horizonte, MG, Brasil.

**Keywords:** Cateteres venosos centrais, Infecções relacionadas a cateter, Termometria, Raios infravermelhos, Diagnóstico precoce, Terapia intensiva

## Abstract

**Objetivo:**

Avaliar se a temperatura da superfície corporal nas proximidades da área de inserção do cateter venoso central é diferente quando os pacientes desenvolvem infecções da corrente sanguínea relacionadas ao cateter.

**Métodos:**

Estudo transversal observacional. Usando um termômetro infravermelho sem contato, 3 medições consecutivas de temperatura da superfície corporal foram coletadas de 39 pacientes com cateter venoso central nos seguintes locais: nas proximidades da área de inserção do cateter ou do reservatório do cateter totalmente implantável, na região contralateral equivalente (sem cateter), e na fronte do mesmo paciente.

**Resultados:**

Um total de 323 observações foram coletadas. Respectivamente nos pacientes do sexo masculino e feminino, desconsiderando a ocorrência de infecção, a temperatura média na área do cateter menos a da região contralateral (média ± desvio padrão: -0,3±0,6°C *versus* -0,2±0,5°C; p=0,36) e a da área do cateter menos a da fronte (média ± desvio padrão: -0,2±0,5°C *versus* -0,1±0,5°C; p=0,3) resultaram em valores negativos. Além disso, em pacientes infectados, foram obtidos valores mais elevados na área do cateter (IC95%: 36,6-37,5ºC *versus* 36,3-36,5ºC; p<0,01) e nas subtrações de temperaturas: área do cateter menos região contralateral (IC95%: -0,17 - +0,33°C *versus* -0,33 - -0,20°C; p=0,02) e a área do cateter menos fronte (IC95%:-0,02 - +0,55°C *versus* -0,22 - -0,10ºC; p<0,01).

**Conclusão:**

Utilizando um termômetro infravermelho sem contato, os pacientes com infecções da corrente sanguínea associadas ao cateter apresentaram valores de temperatura mais elevados, tanto ao redor da área de inserção do cateter e na subtração das temperaturas das regiões contralateral e fronte, em relação àquelas da área do cateter.

## INTRODUÇÃO

Os cateteres venosos centrais (CVC) são dispositivos que permitem a administração de medicamentos e a tomada de medidas hemodinâmicas que não poderiam ser realizadas com segurança de outra forma.^1^ Esses acessos vasculares podem ser classificados como de curta e longa permanências. Os CVC de curta permanência são geralmente cateteres de poliuretano, não tunelizados, com um único lúmen ou múltiplos lúmens e que devem ser inseridos somente em pacientes hospitalizados para uso durante alguns dias ou semanas.^2^ Alguns exemplos de CVC de longa permanência (>3 meses) são os cateteres tunelizados com *cuff* (por exemplo, de HICKMAN^®^ ou BROVIAC^®^) e os do tipo Port, totalmente implantados. Esses últimos são os preferidos para pacientes que necessitam de acesso vascular intermitente.^3^

Aproximadamente 5 milhões de CVC são inseridos por ano, nos Estados Unidos, com uma taxa documentada de infecções relacionadas variando de 3 a 26% por ano.^4^ ^-^ ^7^ Se considerarmos somente as unidades de terapia intensiva, 80 mil infecções da corrente sanguínea relacionadas a cateteres (ICSRC) ocorreram em 1 ano.^8^ Os custos associados a cada caso de ICSRC podem atingir US$56.000^9^, com um aumento médio de 6,5 dias no período de internação para os pacientes mais graves.^10^ Além disso, as ICSRC associam-se a até 25% de mortalidade,^8^ ^,^ ^10^ à substituição de cateter e à interrupção de terapia intravenosa.^11^ ^,^ ^12^

Alguns pesquisadores descreveram uma baixa sensibilidade para detecção de inflamação local em torno da área de inserção do CVC com base em achados clínicos (dor, eritema, edema e purulência).^13^ Relataram também que a colonização do dispositivo vascular pode ocorrer até mesmo na ausência de sintomas e sinais de infecção.^14^ ^,^ ^15^ Por outro lado, a temperatura da superfície corporal, medida com um termômetro infravermelho sem contato (NCIT, *non-contact infrared thermometer*) pode estar alterada nas proximidades de processos infecciosos.^16^

Por esse motivo, parece razoável levantar a hipótese de que o NCIT possa ser útil para o diagnóstico precoce de ICSRC.

## OBJETIVO

Avaliar se a temperatura da superfície corporal na região de inserção do cateter venoso central é diferente quando os pacientes desenvolvem infecções relacionadas ao cateter, quando medida por um termômetro infravermelho sem contato.

## MÉTODOS

### Tipo de estudo e pacientes

Trata-se de um estudo transversal observacional com o objetivo de descrever a temperatura na superfície corporal, medida com um NCIT, na proximidade do local de inserção de CVC não tunelizados, ou onde o reservatório do CVC totalmente implantável ficou posicionado. Foram incluídos pacientes internados nas enfermarias de clínica médica, ou na unidade de terapia intensiva de um hospital universitário público, que necessitavam de um CVC para cuidados médicos. Por outro lado, foram excluídos pacientes em tratamento de ICSRC no início da coleta dos dados. O estudo foi aprovado pelo Comitê de Ética da instituição, e todos os indivíduos ou seus parentes assinaram um Termo de Consentimento Livre e Esclarecido.

### Instrumentos de avaliação

Utilizou-se um NCIT (Extech, modelo IR200^®^, Waltham, Massachusetts, Estados Unidos), com emissividade fixa de 0,95, acurácia de ±0,3°C (±0,5°F) e resolução de 0,1°F/°C, capaz de medir a temperatura corporal de 32 a até 42,5°C (89,6 a 108,5°F). As medidas foram obtidas ao menos 15 minutos após banho, mobilização, ou colocação de compressas quentes ou frias sobre a superfície corporal dos pacientes, nas seguintes áreas: (1) próximo à inserção do CVC não tunelizado ou do reservatório do CVC totalmente implantável; (2) na região contralateral equivalente; e (3) na fronte. Para evitar que uma única medição pudesse resultar em valores atípicos, três medições consecutivas foram aferidas em cada área mencionada acima, e o valor médio foi registrado. O mesmo pesquisador realizou todas as medições. Durante as medições, a distância entre o NCIT e a pele do paciente foi de 10cm. A área-alvo ficou dentro de um raio de 3cm, medialmente à inserção do cateter ou ao reservatório do cateter totalmente implantado. Todas as medições foram feitas até a remoção do cateter sem o conhecimento do estado de infecção dos pacientes. Além disso, o diagnóstico de ICSRC foi confirmado por análise de prontuários médicos após a alta ou o óbito do paciente.

Suspeita de ICSRC foi definida na presença de febre, calafrios e hipotensão, e na ausência de hipovolemia ou doença cardíaca.^17^ Em todos os pacientes com suspeita, o diagnóstico foi estabelecido de acordo com critérios laboratoriais,^18^ confirmados por meio de cultura quantitativa positiva da ponta do cateter ou de hemoculturas quantitativas simultâneas coletadas através do CVC e da veia periférica.^17^

### Análises estatísticas

Inicialmente, comparamos as observações dos pacientes com e sem ICSRC.

Posteriormente, para os pacientes com suspeita de ICSRC, os dados coletados no intervalo de 3 dias antes e 1 dia depois que as hemoculturas tornaram-se positivas foram associadas à ICSRC. Depois, foram feitas comparações entre aqueles com ICSRC confirmada e aqueles em que a ICSRC foi descartada.

As estatísticas descritivas, na forma de média, desvio padrão (DP), variação (mínimo - máximo), e porcentagem (%) foram utilizadas para avaliar as características dos pacientes ou das observações. Como este foi o primeiro estudo a explorar o uso do NCIT na ICSRC, escolhemos descrever os achados conforme o sexo. A distribuição normal dos dados e a presença de valores atípicos foram verificadas por meio dos gráficos de probabilidade normal e de caixa. Os dados com distribuição normal tiveram os intervalos de confiança de 95% (IC95%) divulgados e teste de Wilcoxon ou teste *t *de Student foram utilizados, quando adequados. Todos os valores de p relatados são bilaterais. As análises foram realizadas com o STATA/IC, versão 11.2 (StataCorp LP, Estados Unidos). O poder estatístico (1-β) foi determinado com o G*POWER^®^, versão 3.1.9.2 (Universität Kiel, Alemanha).^19^Para todas as análises, o nível de significância foi fixado em 5%.

## RESULTADOS

### Dados descritivos

Entre agosto e novembro de 2011, foram coletados dados de 39 pacientes. A média de idade foi 57 anos [DP: ±17 (16-87); IC95%: 51-63] para ambos os sexos. Para homens, a média de idade foi 58 anos [DP: ±17 (16-78); IC95%: 50-65], e para mulheres, 57 anos [DP:±19 (19-87); IC95%: 47-67]. Outras características dos indivíduos estão na tabela 1.

**Tabela 1:** Características dos 39 voluntários

Variável	n (%)
Idade, anos	
≤65	22 (56)
>65	17 (44)
Sexo	
Masculino	23 (59)
Feminino	16 (41)
Unidade de internação	
Clínica	21 (54)
UTI	18 (46)
Tipo de CVC	
CVC totalmente implantável	9 (23)
CVC não tunelizado	30 (77)
Número de lúmens	
Um	10 (26)
Dois	27 (69)
Três	2 (5)
Veias preferidas para inserção de CVC	
Subclávia direita	15 (38)
Jugular interna direita	15 (38)
Outras	9 (24)
Usos mais comuns de CVC	
Apenas NPT	10 (26)
NPT/fluidos/medicamentos	10 (26)
Outros	19 (48)

UTI: unidade de terapia intensiva; CVC: cateter venoso central; NPT: nutrição parenteral total.

Considerando os valores médios das três medições realizadas em cada área, foram coletadas, ao todo, 323 medições nos pacientes: 192 (59%) nos indivíduos do sexo masculino e 131 (41%) no feminino. Apesar de a distribuição dos pacientes entre as enfermarias clínica e a unidade de terapia intensiva ter sido semelhante, aproximadamente 75% de todas as medições foram obtidas em pacientes clínicos e 25% naqueles internados em unidades de terapia intensiva (Tabela 1). Três quartos de todos os dados foram provenientes de indivíduos com idade inferior a 65 anos de idade, e um quarto dos dados vieram de pacientes mais velhos. Em 55% das observações, havia um CVC de lúmen único e, em 43%, de dois lúmens. Cinco das medições (aproximadamente 2%) foram realizadas em dois pacientes com cateteres de lúmen triplo.

Essa distribuição não foi semelhante nos dois sexos, tendo sido cateteres de duplo lúmen mais observados em pacientes do sexo feminino (60%). Da mesma forma, em geral, a quantidade de medições relacionadas aos pacientes com CVC não tunelizados e CVC totalmente implantáveis, respectivamente, ficou distribuída em proporções quase iguais (49% e 51%), porém, de forma diferente quando a análise foi realizada por sexo (42% e 58% nos homens e 59% e 41% nas mulheres). Como nosso hospital é uma unidade de referência no tratamento de pacientes com insuficiência intestinal, até 65% dos dados foram coletados em pacientes com a síndrome do intestino curto. Esses pacientes são internados com uma frequência maior para receberem nutrição parenteral total (NPT), já que a nutrição parenteral domiciliar não está disponível no sistema público de saúde brasileiro.

As temperaturas na área do cateter (e também no reservatório totalmente implantável), na região contralateral e na fronte foram diferentes entre os sexos (Tabela 2). Ademais, a subtração das temperaturas da área do cateter menos aquelas do lado contralateral resultou em valores médios negativos tanto nos homens como nas mulheres, respectivamente (média±DP: -0,3±0,6 *versus* -0,2±0,5°C; p=0,36). Um achado semelhante foi observado ao se subtrair a temperatura da fronte das temperaturas da área do cateter (média±DP: -0,2±0,5 *versus* -0,1±0,5°C; p=0,3).

**Tabela 2:** Medições de temperatura por sexo

	Masculino	Feminino	Valor de p*
	(n=192)	(n=131)	
Área da temperatura (ºC)			
Área do CVC	36,4±0,7 (34,9 - 38,2)	36,5±0,8 (34,5 - 39)	0,01
Região contralateral	36,6±0,6 (32,2 - 38,3)	36,8±0,7 (34,5 - 38,2)	0,02
Fronte	36,5±0,5 (35,2 - 38,1)	36,7±0,6 (34,3 - 37,9)	<0,01

Subtrações (ºC)			
CVC – contralateral	-0,3±0,6 (-1,9 - +3,1)	-0,2±0,5 (-1,5 - +1,3)	0,36
CVC – fronte	-0,2±0,5 (-1,7 - +1,3)	-0,1±0,5 (-1,5 - +1,4)	0,3

Os dados estão expressos em média ± desvio padrão; variação (mínima - máxima). * valores de p calculados com teste de Wilcoxon e *t *de Student’s, conforme apropriado (α=5%). CVC: cateter venoso central.

### Principais resultados do estudo

Quatro pacientes, dois de cada sexo, tiveram seis casos de infecção relacionados ao CVC durante o estudo (dois pacientes tiveram dois casos). Todos tinham idade inferior a 65 anos, nenhum estava em unidade de terapia intensiva e três tinham um CVC totalmente implantável. Os patógenos identificados foram *Acinetobacter junii*, *Staphylococcus aureus* resistente à meticilina e *Staphylococcus warneri*. Aproximadamente 5% (n=15) das observações foram colhidas com um intervalo de 3 dias antes e 1 dia depois das hemoculturas positivas terem sido colhidas. Descartando duas observações (valores atípicos) e comparando as medições restantes com as observações não relacionadas à ICSRC, foram encontrados valores maiores em pacientes com infecção nas duas subtrações de temperatura: área do cateter menos região contralateral (IC95%: -0,17 - +0,33 *versus *-0,33 - -0,20°C; p=0,02; poder estatístico de 0,82), e área do cateter menos fronte (IC95%: -0,02 - +0,55* versus *-0,22 - -0,10°C; p<0,01; poder estatístico de 0,88), respectivamente. Além disso, a temperatura da área do cateter foi mais alta em pacientes com ICSRC (IC95%: 36,6-37,5 *versus* 36,3-36,5°C; p<0,01; poder estatístico de 0,94) (Tabela 3).

**Tabela 3:** Temperatura (ºC) nas observações relacionadas ou não a infecção

	Relacionadas a infecção		Não relacionadas a infecção		Valor de p*
	(n=13)		(n=308)		
	Média±DP	IC95%	Média±DP	IC95%	
Área do CVC	37,1±0,7	36,6 - 37,5	36,4±0,7	36,3 - 36,5	<0,01
Região contralateral	37,0±0,6	36,6 - 37,3	36,7±0,7	36,6 - 36,8	0,12
Fronte	36,8±0,6	36,4 - 37,1	36,6±0,6	36,5 - 36,6	0,20
CVC – contralateral	+0,08±0,42	-0,17 - +0,33	-0,27±0,54	-0,33 - -0,2	0,02
CVC – fronte	+0,27±0,48	-0,02 - +0,55	-0,16±0,52	-0,22 - -0,1	<0,01

* Valores de p calculados com teste *t *de Student’s (α=5%). DP: desvio padrão; IC95%: intervalo de confiança de 95%; CVC: cateter venoso central.

Entre as 308 observações não associadas a hemoculturas positivas, 16 foram coletadas na presença de sinais clínicos sugestivos de infecção, entre 3 dias antes e 1 dia após a coleta das hemoculturas negativas. Assim, quando as medições relacionadas às hemoculturas negativas e positivas foram comparadas, a temperatura ao redor da área de inserção do CVC foi mais alta naqueles com ICSRC (p=0,03; poder estatístico de 0,58). Ademais, valores médios maiores foram encontrados em pacientes com infecção nas duas subtrações de temperaturas: área do cateter menos fronte (p=0,03; poder estatístico de 0,85) e área do cateter menos região contralateral (p=0,02; poder estatístico de 0,45), respectivamente (Tabela 4).

**Tabela 4:** Temperatura (ºC) nas observações relacionadas a hemoculturas

	Hemoculturas positivas	Hemoculturas negativas	Valor de p^*^
	(n=13)	(n=16)	
	Média±DP	Média±DP	
Área do CVC	37,1±0,7	36,5±0,5	0,03
Região contralateral	37,0±0,6	36,7±0,2	0,21
Fronte	36,8±0,6	36,7±0,2	0,16
CVC – contralateral	+0,08±0,42	-0,27±0,48	0,02
CVC – fronte	+0,27±0,48	-0,22±0,46	0,03

^* ^Valores de p calculados com teste de Wilcoxon (α=5%). DP: desvio padrão; CVC: cateter venoso central.

## DISCUSSÃO

Os NCITs foram introduzidos em alguns aeroportos em áreas de aglomeração durante o surgimento da síndrome respiratória aguda grave (SARS) em 2003, com relatos de baixa eficácia.^20^ Ao mesmo tempo, alguns estudos sugeriram que o NCIT poderia ser uma alternativa confiável para medição da temperatura corporal, principalmente em pacientes pediátricos.^21^ ^-^ ^23^ Seu princípio é de que o calor emitido pelo corpo pode ser detectado no espectro de radiação infravermelha por meio de um sensor remoto.^24^

Até onde se sabe, este é o primeiro estudo sobre termometria infravermelha e infecções relacionadas a cateteres. Durante a coleta de dados, seis casos de ICSRC foram confirmados em quatro pacientes, principalmente naqueles com a síndrome do intestino curto que receberam NPT e outros medicamentos ou fluidos através de um CVC totalmente implantável. Duas das 15 observações relacionadas a hemoculturas positivas geraram resultados negativos pronunciados (valores atípicos) a partir da subtração de temperaturas da região contralateral daquelas da área do CVC (-1,3°C e -1,4°C). Quando essas duas medições foram feitas, não havia qualquer infusão sendo realizada pelo cateter e o paciente estava recebendo um tratamento com* lock* com etanol a 70% através do cateter.^25^ No entanto, não há garantia de sua relação com os resultados obtidos. Após a exclusão dessas duas medições, foram observadas diferenças entre as subtrações de temperaturas da área do CVC menos aquelas da região contralateral e da fronte, comparando-se os dados relacionados e sem ICSRC. A diferença observada somente em torno da área de inserção do CVC dos pacientes infectados indica que, quando ocorre a ICSRC, a temperatura da superfície corporal, medida com um NCIT, em torno da área de inserção do CVC ou de seu reservatório, torna-se mais alta.

Quando as 13 observações, coletadas no período de 3 dias antes e 1 dia após as hemoculturas positivas, foram comparadas com as 16 medições do mesmo período, porém, após hemoculturas negativas, resultados semelhantes foram observados.

Dentre os pacientes com suspeita de infecção e dos quais foram colhidas culturas sanguíneas, foi encontrado um baixo poder estatístico quando comparados os valores da área do CVC e da subtração da área do CVC menos a região contralateral. O pequeno número de observações relacionadas a hemoculturas poderia ser uma explicação.^26^

Recentemente, estudos destacaram os usos diagnósticos e prognósticos das medições de temperatura para doenças infecciosas. Padrões anormais de temperatura corporal, avaliados por meio de termômetros comuns, foram revelados como preditivos de sepse.^27^ Além disso, as medições de temperatura da superfície da pele por um NCIT parecem ser um indicador promissor da gravidade, ou da melhoria de uma infecção da pele ou dos tecidos moles.^16^

Em geral, desconsiderando a ocorrência de infecção, foram observados resultados negativos quando as temperaturas das regiões contralateral e da fronte foram subtraídas daquelas obtidas na área do cateter/reservatório. Uma temperatura mais baixa na área do cateter poderia ser explicada pela infusão quase que contínua de fluidos (isto é, nutrição parenteral e soro fisiológico), os quais normalmente são mantidos abaixo da temperatura corporal. Uma infusão de 2L de cristaloide em temperatura ambiente diminui o calor do corpo em um terço, em graus Celsius.^28^ No entanto, esse pressuposto deve ser testado em estudos futuros.

Com relação às limitações do estudo, a quantidade de observações não foi igualmente obtida entre os pacientes, por três motivos: (1) o tempo de hospitalização variou entre eles devido a óbito, melhora ou piora do quadro; (2) a coleta de dados não estava disponível para alguns pacientes em determinado dia devido à realização dos procedimentos; (3) as medições de temperatura foram feitas somente em dias úteis. Portanto, aproximadamente 41% das medições estavam relacionadas a somente 3 pacientes, enquanto outros 18 indivíduos contribuíram com menos de 1% do total. Além disso, não foram investigadas mudanças no calor corporal causadas pela temperatura do fluido infundido no momento da coleta de dados. Finalmente, este estudo não avaliou as influências da gravidade da doença, da temperatura ambiente e da estabilidade hemodinâmica sobre os resultados, considerando que a temperatura da superfície corporal em determinadas áreas é diferente para cada tipo de dispositivo vascular.

Estudos adicionais precisam confirmar esses achados. Além disso, amostras maiores parecem ser necessárias para detectar diferenças entre pacientes com suspeita de infecção com maior poder estatístico.^29^ Finalmente, a acurácia diagnóstica^30^ precisa ser abordada.

## CONCLUSÃO

Utilizando um termômetro infravermelho sem contato, em pacientes com infecções da corrente sanguínea relacionadas a cateteres foram observados valores de temperatura mais altos na área de inserção do cateter venoso central e por meio das subtrações entre as temperaturas das regiões da área do cateter, contralateral e da fronte.
